# Sorption and Release of Organics by Primary, Anaerobic, and Aerobic Activated Sludge Mixed with Raw Municipal Wastewater

**DOI:** 10.1371/journal.pone.0119371

**Published:** 2015-03-13

**Authors:** Oskar Modin, Soroush Saheb Alam, Frank Persson, Britt-Marie Wilén

**Affiliations:** Division of Water Environment Technology, Department of Civil and Environmental Engineering, Chalmers University of Technology, Gothenburg, Sweden; Purdue University, UNITED STATES

## Abstract

New activated sludge processes that utilize sorption as a major mechanism for organics removal are being developed to maximize energy recovery from wastewater organics, or as enhanced primary treatment technologies. To model and optimize sorption-based activated sludge processes, further knowledge about sorption of organics onto sludge is needed. This study compared primary-, anaerobic-, and aerobic activated sludge as sorbents, determined sorption capacity and kinetics, and investigated some characteristics of the organics being sorbed. Batch sorption assays were carried out without aeration at a mixing velocity of 200 rpm. Only aerobic activated sludge showed net sorption of organics. Sorption of dissolved organics occurred by a near-instantaneous sorption event followed by a slower process that obeyed 1^st^ order kinetics. Sorption of particulates also followed 1^st^ order kinetics but there was no instantaneous sorption event; instead there was a release of particles upon mixing. The 5-min sorption capacity of activated sludge was 6.5±10.8 mg total organic carbon (TOC) per g volatile suspend solids (VSS) for particulate organics and 5.0±4.7 mgTOC/gVSS for dissolved organics. The observed instantaneous sorption appeared to be mainly due to organics larger than 20 kDa in size being sorbed, although molecules with a size of about 200 Da with strong UV absorbance at 215–230 nm were also rapidly removed.

## Introduction

Modern wastewater treatment is dominated by the activated sludge process, which was developed over 100 years ago [1]. Organic compounds are biologically oxidized in an aerated tank. Then, the sludge is typically separated from the treated effluent in sedimentation tanks, partly returned to the inlet of the aerated tank, and partly wasted as excess sludge (or waste activated sludge, WAS) [2]. Alternatively, membrane filtration can be used to separate the activated sludge from the treated water [3].

In many existing and emerging activated sludge process configurations, rapid sorption of organic compounds from the wastewater onto the sludge plays an important role for the removal. Existing process configurations that rely on sorption as a major removal mechanism include contact-stabilization [4] and adsorption-biooxidation (AB) [5,6]. In the contact-stabilization process, the influent wastewater is mixed with activated sludge in a contact tank having a short hydraulic retention time (HRT) of e.g. 15 min. Organic compounds are assumed to rapidly sorb onto the sludge, which is separated from the treated water in a sedimentation tank. The settled sludge is then aerated in a stabilization tank to oxidize the sorbed organics, before being recycled back to the contact tank. The AB process is a two-sludge system consisting of a high-rate activated sludge process (the A-stage) operated with a short solids retention time (SRT) of 3–12 hours. In the A-stage, organics are removed mainly by sorption onto the sludge. The low-loaded B-stage is then used e.g. for nutrient removal and oxidation of the organic compounds remaining in the wastewater after the A-stage [7,8].

Directing WAS to the primary settlers to be mixed with the primary sludge in a wastewater treatment plant is another quite common practice to utilize the sorptive capacity of the WAS and improve the dewatering properties of secondary sludge [2,9,10]. However, scientific studies on the effect of activated sludge addition on organics removal in primary sedimentation are scarce. Yetis and Tarlan [9] found that addition of WAS improved sedimentation of suspended solids in raw wastewater under certain conditions. Tests were carried out with sludge cultivated at different solids retention times and generally concentrations above 1600 mg total suspended solids (TSS)/L gave the best results. However, no information was provided about dissolved substances. Ross and Crawford [11] carried out full-scale tests comparing primary settlers that either received or did not receive WAS. However, they did not see any significant differences in the organics content in the effluent from the settlers.

New activated sludge processes based on sorption as the major mechanism for organics removal are also emerging. As energy-efficiency and carbon footprint are becoming more and more important for wastewater treatment plants [12], high-rate activated sludge processes similar to the A-stage of an AB process are being investigated [13,14]. A high-rate activated sludge process with short solids retention time (SRT) is potentially more energy-efficient than a low-rate process with long SRT because less oxygen is needed per mass of organic material removed and more excess sludge is produced, which can be converted to biogas in an anaerobic digester. Thus, a high-rate process could both cut electricity consumption because of lower aeration requirements and increase energy output (in the form of produced biogas) for a wastewater treatment plant [15].

Sorption-based processes have also been investigated as an enhanced form of primary treatment. Huang and Li [16] recycled primary sludge and mixed it with the raw wastewater before primary sedimentation. Enhanced organics removal could only be obtained after the primary sludge had been aerated in a stabilization tank. A COD removal efficiency of 40% could be obtained which was 35% higher than with primary sedimentation alone. Zhao et al. [17] investigated a bioflocculation-adsorption, sedimentation and stabilization process for enhanced primary treatment. They obtained total COD removal efficiencies of 70–80%. Both of the processes referred to here are very similar to the contact-stabilization process.

To design and predict the performance of sorption-based activated sludge processes for enhanced primary treatment or for more energy-efficient wastewater treatment, studies investigating the sorption capacity of sludge are needed. The goal of this study is to quantify the sorption of particulate and dissolved organic compounds onto different types of sludge available at wastewater treatment plants. We compare primary, anaerobic, and aerobic activated sludge as sorbents for wastewater organics. For aerobic activated sludge, we also investigate the effect of starvation on sorption capacity, determine sorption kinetics, and investigate some characteristics of the sorbed organics.

## Materials and Methods

### Collection of sludge and wastewater

Activated sludge, primary sludge, anaerobic digester sludge, raw municipal wastewater, and treated effluent samples were collected at the Rya wastewater treatment plant, which treats about 4.4 m^3^/s of municipal wastewater from the city of Gothenburg, Sweden. Permission to take samples at the plant was granted by Gryaab. The activated sludge basins at the plant have a solids retention time of 3–5 days and consist of an anoxic pre-denitrification zone followed by an aerobic zone. The basins are aerated using a diffused aeration system. Activated sludge samples were collected near the outlet of the aerobic zone. Primary sludge refers to sludge collected from the primary settlers. Anaerobic sludge was collected from a mesophilic digester treating sludge generated at the plant. The raw municipal wastewater refers to the influent to the plant before it had passed any treatment steps. The treated effluent refers to the final effluent from the plant collected after disc filtration, which is the final treatment operation. Starved activated sludge, which was used in some experiments, was obtained by keeping 8-L activated sludge in an aerated tank stirred at 200 rpm using a 4-bladed propeller (10 cm diameter) for 1, 3 and 6 days without the addition of substrate. All experiments and analyses were carried out less than 8 hours from the time samples were collected at the plant.

### Sorption tests

In the sorption tests, 100 mL of sludge suspension was mixed with 600 mL of raw municipal wastewater in 1-L beakers containing paddles (width 5.5 cm, height 3 cm) stirring the mixtures at 200 rpm. The velocity gradient in the beakers was estimated to 4.3 s^-1^ (the calculation is described in S1 File). After 5 min, mixing was stopped and the sludge-wastewater mixtures were allowed to settle for 30 min. Then, approximately 100 mL of the supernatant was collected for analyses. The sludge suspensions were prepared by centrifugation of the desired sludge volume at 1300g, decantation of the supernatant, and resuspension in 100 mL of effluent water. In control tests, 100 mL of effluent without added sludge was added to the 600 mL of wastewater. Measurements of conductivity, total suspended solids (TSS) and volatile suspended solids (VSS) concentrations, sludge volume (SV) and sludge volume index (SVI), particulate organic carbon (TOCp) and dissolved organic carbon (TOCd) concentrations, and absorbance measurements at 650 nm (ABS650) and 254 nm (ABS254) were carried out to characterize the samples and measure removal of organic compounds. All experiments were carried out in room temperature (about 22°C).

### Statistical methodology for the sorption tests

On each sampling day, the removal efficiencies of particulate and dissolved organic carbon were compared under three conditions: (1) addition of treated effluent (control), (2) addition of low concentration of activated sludge (0.30–0.39 gTSS/L), and (3) addition of high concentration of activated sludge (1.04–1.27 gTSS/L). The concentrations of added sludge refer to the final concentrations in the wastewater and sludge suspension mixtures. Duplicate tests were carried out for each condition. Paired-sample t-tests were conducted on the removal efficiencies for the different conditions. The null hypothesis was that there would be no difference in the removal efficiency with and without sludge addition. The null hypothesis was rejected if the two-tailed p-value was less than 0.05.

### Kinetic tests

To determine the rate of sorption, kinetic tests were carried out using similar conditions to the sorption tests described above. After 1, 5, 10, 15, 30, 60, and 120 min of mixing, 50 mL of solution was withdrawn from each beaker and allowed to settle for 30 min in a separate vial. A sample of the supernatant was analyzed for ABS650 as a proxy for TSS concentration. A centrifuged sample (5 min at 4000g) was analyzed for ABS254 as a proxy for TOCd concentration [18]. Repeated measurements of ABS650, ABS254, TSS, and TODd of several wastewater and sludge samples from seven different sampling occasions showed strong correlations between ABS650 and TSS concentration (R^2^ = 0.99), and ABS254 and TOCd concentration (R^2^ = 0.94) (S2 File). On one of the test days, high-performance size exclusion chromatography (HPSEC) was carried out to investigate the fate of organic molecules of the size range 100–20 000 Da during the kinetic sorption tests.

The sorption was modelled using 1^st^ order kinetics, which has previously been observed to describe sorption of particulate chemical oxygen demand (COD) onto activated sludge [19] (Equation 1).

dC/dt=−kxXx(C−a) (1)

where *dC/dt* is the rate of change of organic sorbate concentration, *C*, with time, *t* (mg/L·min), *k* is the first order rate constant (L/mgVSS·min), *a* is the residual concentration (mg/L), and *X* is the activated sludge concentration (mgVSS/L).

The rate constant, *k*, and residual concentration, *a*, were found using Excel Solver by fitting the experimental data to Equation 2.

$$C(t)=a+(C_{0}-a)xexp(-kxXxt)$$ (2)

where *C(t)* is the organic sorbate concentration at time *t*, and *C*_*0*_ is the initial concentration.

### Azide inhibition tests

To quantify the effect of microbial respiration on the measured sorption rates of dissolved organics, experiments with sodium azide-inhibited sludge was carried out. The experiments were performed in a similar way as the kinetic tests with sampling after 1, 5, 10, 15, 30, and 60 min of mixing. Before the experiments, the sludge was exposed to approximately 0.2 gNaN_3_/gTSS for three hours [20], which is a load known to inhibit the respiratory activity of activated sludge [21]. Sodium azide can cause deflocculation of sludge; therefore, controls with addition of azide-inhibited sludge to beakers containing treated effluent were run in parallel to quantify the release of organic substances by the sludge. Controls with live activated sludge were also run in parallel to allow direct comparison between azide-inhibited and live sludge.

### Analytical methods

Conductivity was measured using a probe (WTW TetraCon325). TSS and VSS concentrations were analyzed according to Standard Methods [22] using glass-fiber filter papers (Munktell, grade MGA). SV was analyzed by allowing the sludge to settle in a 1-L graduated cylinder and measuring the volume occupied by the settled sludge after 30 min. SVI was obtained by dividing the SV by the TSS concentration. TOCp and TOCd concentrations were analyzed by a total organic carbon analyzer (TOC-V, Shimadzu). The TOC was divided into TOCp and TOCd fractions based on filtration through a 0.45 μm membrane-filter. Cake layer formation on the membrane during filtration could potentially result in retention of smaller particles than 0.45 μm. To minimize this effect, samples were centrifuged for 2 min at 4000 g before being filtered. Absorbance at 650 nm (ABS650) and at 254 nm (ABS254) was measured with a spectrophotometer (UV-1800, Shimadzu). Specific UV absorbance (SUVA) was calculated as the ratio between ABS254 and TOCd concentration. SUVA is an indicator of the aromatic content of the dissolved organic carbon [23]. HPSEC was carried out using a Shimadzu HPLC system equipped with UV and refractive index detectors, an Agilent Bio SEC-5 column (length 300 mm, diameter 7.8 mm, pore size 100Å), and a mobile phase made up of 100 mM NaCl, 8.3 mM KH_2_PO_4_, and 11.7 mM K_2_HPO_4_ being pumped at 0.5 mL/min. The molecular sizes of the eluted molecules were calibrated against retention time using polyethylene glycol standards (PEG-10 calibration kit, Agilent). The organic compounds in the sorption test samples were analyzed using the UV detector at wavelengths of 215, 230, 254, and 280 nm. Low molecular weight carboxylic acids (C1–C5) were analyzed using the same HPLC system equipped with an Aminex HPX-87H column (BioRad) and operated with a mobile phase of 5 mM H_2_SO_4_ pumped at 0.5 mL/min. The acids were detected at 210 nm.

## Results

### General

Tests were carried out with wastewater and sludge collected at the treatment plant on eight different days. In the influent wastewater, the conductivity ranged from 791 to 1022 μS/cm and the TSS concentration ranged from 0.109 to 0.926 g/L. The activated sludge had a TSS concentration of 2.08–2.73 g/L, a VSS/TSS ratio of 70–73%, and a SVI of 70–96 mL/gTSS. The primary sludge and anaerobic digester sludge had TSS concentrations of approximately 47 g/L and 30 g/L, respectively. The treated effluent from the plant contained 11–13 mg/L of TOCd and negligible concentrations of TSS.

### Net release of organics by primary- and anaerobic sludge

**Fig 1:**
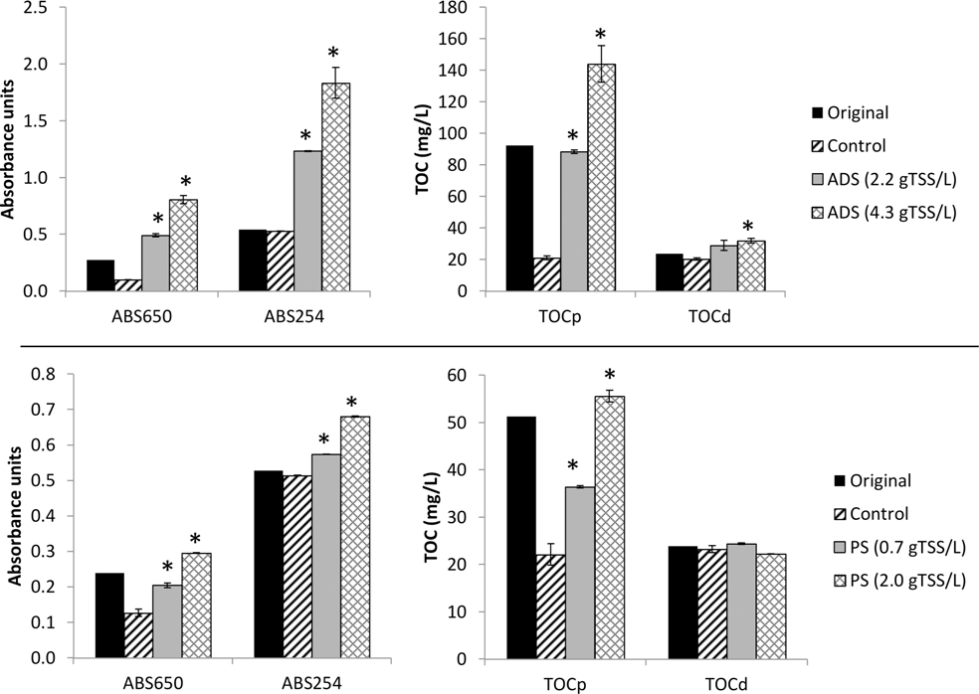
Absorbance at 650 nm and 254 nm and concentrations of TOCp and TOCd in the mixtures of wastewater with effluent (Control), anaerobic digester sludge (ADS) and primary sludge (PS) of different concentrations. The original TOC concentrations and absorbance values before sedimentation are also shown. Averages of duplicate measurements are shown with the error bars representing the individual measurements. An asterisk (*) above a column indicate that the removal efficiency was significantly different (p<0.05, n = 2) from the control.

The original concentrations of particulate and dissolved organics and the final concentration after 5 min mixing and 30 min sedimentation in controls and tests with addition of primary- and anaerobic sludge are shown in Fig. 1. Tests with addition of primary sludge and anaerobic digester sludge showed a net release of organic compounds from the sludge into the wastewater. Compared to the controls, both anaerobic digester sludge and primary sludge released particles into the wastewater, which can be seen by the increase in ABS650 and TOCp values. The amount of released TOCp was related to the amount of added sludge. For anaerobic digester sludge it was 45.3–49.5 mgTOCp/gVSS, whereas primary sludge released 21.7–27.8 mgTOCp/gVSS. For dissolved substances, anaerobic digester sludge released 4.3–6.3 mgTOCd/gVSS, whereas primary sludge did not have a net release of TOCd. However, the ABS254 and consequently the SUVA increased with both sludges. This indicates that aromatic organics, such as humic acids, were released by the sludge. In the primary sludge, this release was balanced by an uptake of other organics resulting in no net change in the TOCd concentration.

### Net sorption of organics by aerobic activated sludge

**Fig 2:**
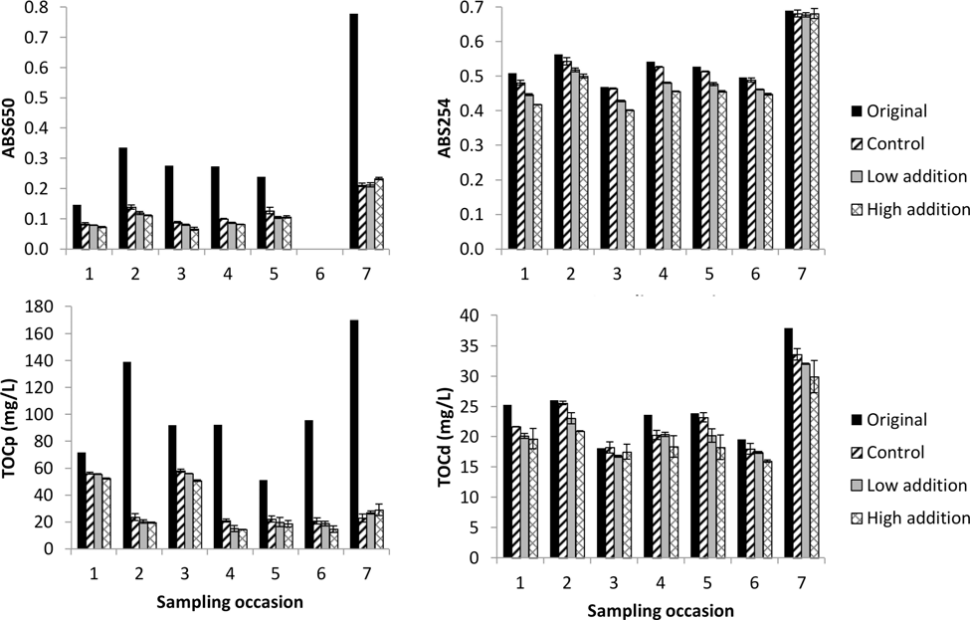
Absorbance at 650 nm and 254 nm and concentrations of TOCp and TOCd in the mixtures of wastewater with effluent or activated sludge suspension. Original refers to the concentrations before sedimentation. Control, low addition, and high addition refers to the concentration in the supernatant after 30 min sedimentation with zero (control), 0.30–0.39 gTSS/L (low addition), or 1.04–1.37 gTSS/L (high addition) activated sludge. Averages of duplicate measurements are shown with the error bars representing the individual measurements. ABS650 was not measured on sampling day 6.

The change in TOCp and TOCd concentrations, ABS254 and ABS650 for the sorption tests carried out with aerobic activated sludge on seven different sampling days are shown in Fig. 2. The influent wastewater characteristics varied between the different sampling days, especially in terms of particulate content (see the original values of TOCp and ABS650 in Fig. 2). Addition of activated sludge generally appeared to have a small but positive impact on the net removal of both particulate (TOCp and ABS650) and dissolved (TOCd and ABS254) organic carbon. To verify that activated sludge addition did indeed have an effect on the removal, statistical analysis was carried out (Table 1). For the parameters ABS650, TOCp, ABS254, and TOCd addition of both low and high concentration of activated sludge always resulted in a statistically significant difference in removal efficiency compared to the controls suggesting that addition of activated sludge could improve removal of both particulate and dissolved substances. For all parameters except ABS650, there was also a significant difference in the removal between low (0.30–0.39 gTSS/L) and high (1.04–1.37 gTSS/L) activated sludge addition (Table 1). The average sorption of TOCp was 6.5±10.8 mgTOCp/gVSS while the sorption of TOCd was 5.0±4.7 mgTOCd/gVSS. Thus, the total organics sorption was 11.5±11.8 mgTOC/gVSS. There was no difference in the SUVA between the controls and the samples with sludge addition suggesting that the aromatic content of the wastewater organics did not change because of mixing with activated sludge.

**Table 1:** Two-tailed p-values for paired-sample t-tests comparing the percentage removal of ABS650, ABS254, TOCp, and TOCd in sedimentation of wastewater without addition activated sludge (control) and with low addition (0.30–0.39 gTSS/L) or high addition (1.04–1.37 gTSS/L).

	ABS650	TOCp	UV254	TOCd
Control vs low addition	**2.2x10** ^**-3**^	**1.6x10** ^**-2**^	**4.6x10** ^**-6**^	**1.1x10** ^**-3**^
Control vs high addition	**2.5x10** ^**-3**^	**1.3x10** ^**-3**^	**5.1x10** ^**-6**^	**2.3x10** ^**-4**^
Low vs high addition	1.3x10^-1^	**1.2x10** ^**-2**^	**3.6x10** ^**-5**^	**3.5x10** ^**-5**^

p-values below 5x10^-2^ are deemed to indicate significant difference in removal and are marked in bold (n = 14).

### Effect of activated sludge starvation

**Fig 3:**
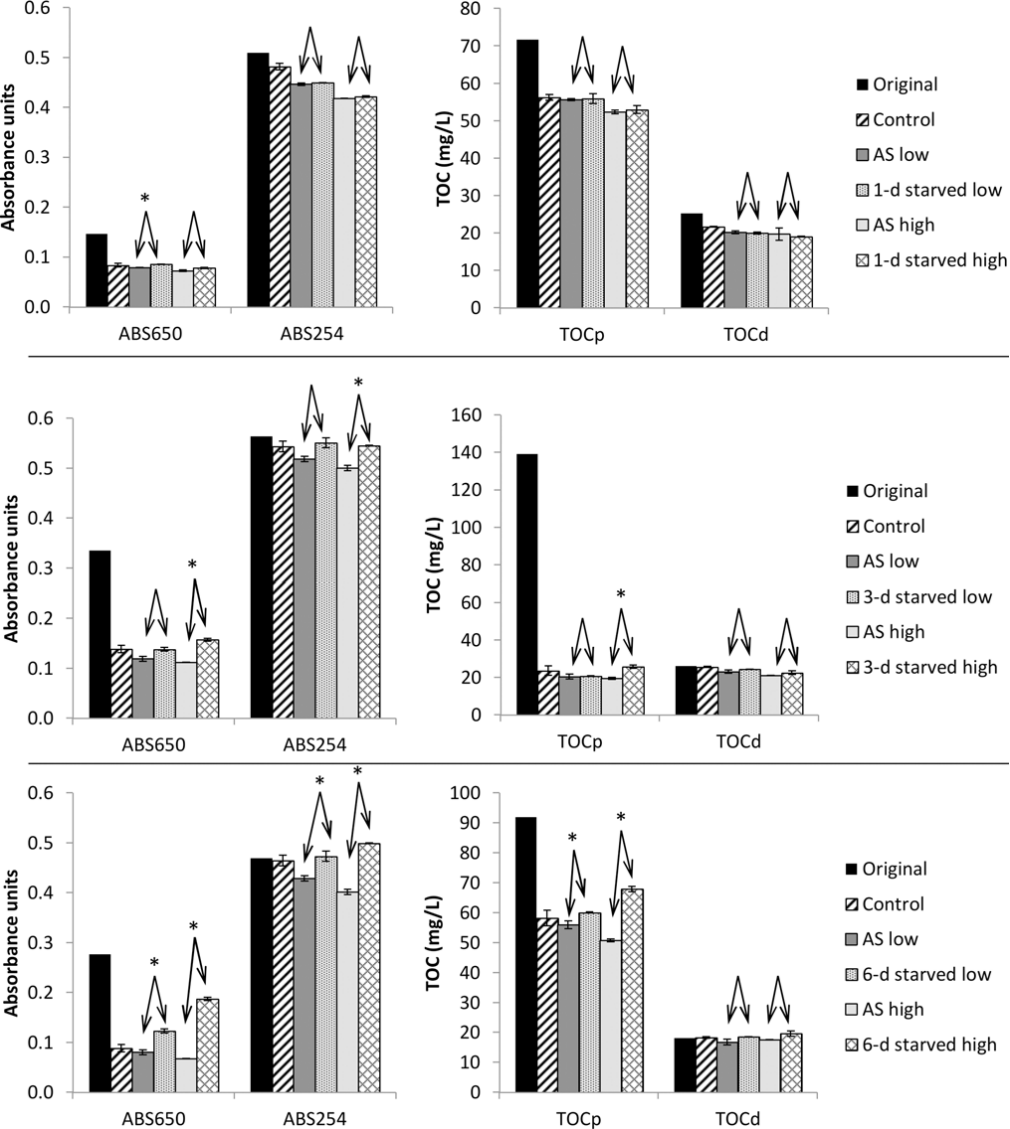
Absorbance at 650 nm and 254 nm and concentrations of TOCp and TOCd in the mixtures of wastewater with effluent (Control), 0.30–0.33 gTSS/L of activated sludge (AS low), 1.04–1.16 gTSS/L of activated sludge (AS high), or activated that had been starved for either 1, 3 or 6 days. The low concentration of the starved sludge was 0.28–0.32 gTSS/L and the high concentration was 0.97–1.13 gTSS/L. The original TOC concentration and absorbance values before sedimentation are also shown. Averages of duplicate measurements are shown with the error bars representing the individual measurements. Arrow pairs indicate that the removal efficiencies for two treatments were compared. An asterisk (*) above the arrow pair indicate statistically significant difference in removal (p<0.05, n = 2).

Starvation of activated sludge may free up adsorption sites, which would allow greater removal of organic compounds from wastewater [24]. Therefore, activated sludge was starved for 1, 3, and 6 days and its sorption ability was compared to fresh sludge collected from the wastewater treatment plant (Fig. 3). After 1 day of starvation, the starved sludge performed approximately equal to the fresh sludge, both resulting in an improvement of the removal of particulate and dissolved substances compared to the control. Only for ABS650 and addition of low concentration of sludge did the starved sludge show significantly worse removal efficiency than the fresh activated sludge. After 3 days starvation, high addition of sludge resulted in significantly lower removal efficiency of ABS650, TOCp, and UV254. After 6 days starvation, addition of both low and high concentrations of starved sludge resulted in significantly worse removal efficiencies than fresh activated sludge for all parameters except TOCd. Under no circumstances did starved sludge perform better than fresh activated sludge. The reason may be that the fresh activated sludge was collected from the outlet of the aeration tanks at the wastewater treatment plant. Thus, organic substances taken up by the sludge in the treatment plant had already been metabolized and further starvation did not free up any additional adsorption sites. Instead, prolonged starvation (> 1 day) led to deflocculation of the sludge and release of particles (ABS650 and TOCp) and humic substances (UV254), which meant that addition of starved sludge to the wastewater had a negative effect on the removal efficiencies of these parameters.

### Sorption kinetics with aerobic activated sludge

**Fig 4:**
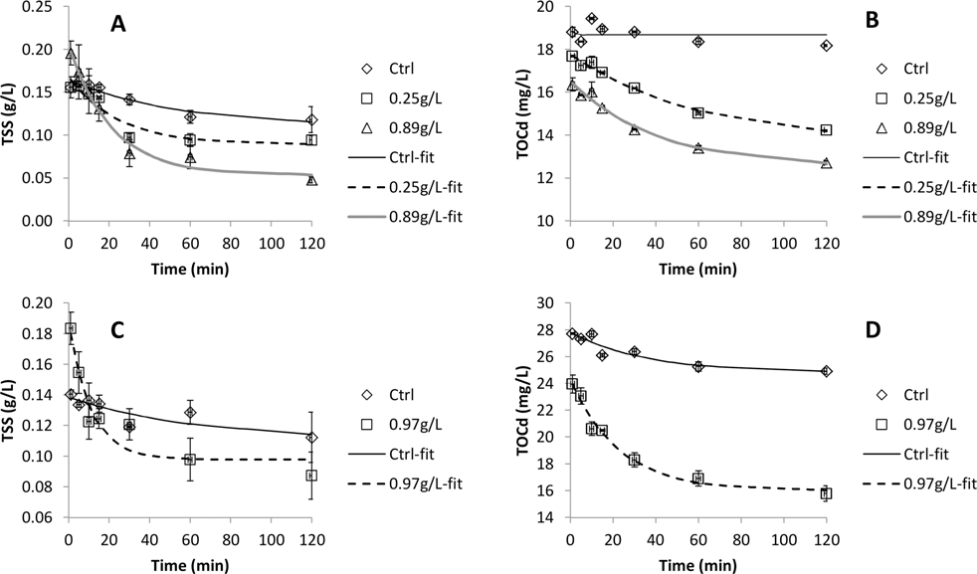
Removal of TSS and TOCd in kinetic tests carried out on two different sampling occasions. The legends show addition of activated sludge in gVSS per litre. Control samples (Ctrl) did not contain added activated sludge. Symbols show measured data whereas lines were fitted using Equation 2. Each measurement point is the average of two replicate tests with the error bars representing the individual measurements.

The sorption kinetics was investigated with raw wastewater and aerobic activated sludge collected on two different days. The changes in concentrations of TSS and TOCd are shown in Fig. 4 while kinetic coefficients and R^2^-values for the fits are shown in Table 2. Upon mixing wastewater and sludge, an instantaneous increase in TSS concentration and decrease in TOCd concentration can be observed. The increase in TSS can be explained by release of small particles by the sludge. The decrease in TOCd suggests an instantaneous sorption of TOCd from the wastewater onto the sludge. Following this instantaneous sorption or release, sorption of both TSS and TOCd obeys 1^st^ order kinetics.

**Table 2:** Instantaneous sorption onto aerobic activated sludge, residual non-sorbable concentration (a), 1^st^ order sorption constant (k), and coefficient of determination (R^2^) for the kinetic tests.

Sample	Instant sorption	a	k	R^2^
***TSS sorption***	(mgTSS/gVSS)	(mgTSS/L)	(L/gVSS.min)	
July 2013: 0.25 gVSS/L	-14	109	0.159	0.90
July 2013: 0.89 gVSS/L	-45	89	0.053	0.98
Oct. 2014: 0.97 gVSS/L	-51	53	0.094	0.91
***TOCd sorption***	(mgTOCd/gVSS)	(mgTOCd/L)	(L/gVSS.min)	
July 2013: 0.25 gVSS/L	8.7	18.7	0.065	0.99
July 2013: 0.89 gVSS/L	3.9	13.6	0.028	0.98
Oct. 2014: 0.97 gVSS/L	3.7	12.5	0.045	0.98
Nov. 2014: 1.51 gVSS/L	2.0–2.1	12.7–13.7	0.014–0.020	0.95–0.98
^a^Nov. 2014: 1.51 gVSS/L	1.0–1.3	18.2–19.1	0.016–0.020	0.82–0.97

^a^Test with azide-inhibited activated sludge.

### Effect of azide inhibition

**Fig 5:**
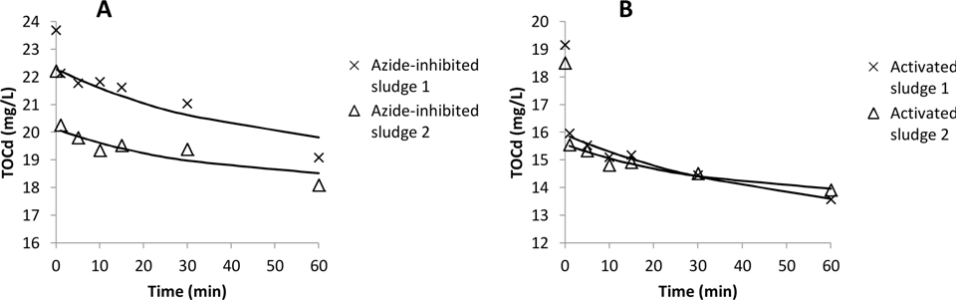
Concentration of TOCd in sorption tests with azide-inhibited (A) and live activated sludge (B). The solid lines show fits using 1^st^-order kinetics for the measurements points between 1 min and 60 min. Two repeated tests were carried out with each type of sludge.

The rate of biosorption of dissolved organics was compared with live and azide-inhibited activated sludge. Azide treatment resulted in some release of organic substances by the sludge, ranging from 2.5 to 3.0 mgTOCd/gVSS in the two tests. This is shown by the higher starting concentrations of TOCd in the sorption tests with azide-inhibited sludge (Fig. 5A) compared to live activated sludge (Fig. 5B). However, both live and azide-inhibited sludge showed a near-instantaneous sorption event upon mixing with raw wastewater. The extent of near-instantaneous sorption was 2.0–2.1 mgTOCd/gVSS for live activated sludge and 1.0–1.3 mgTOCd/gVSS for azide-inhibited sludge. The rate of removal was 24–36 mgTOCd/gVSS∙d for live activated sludge and 30–48 mgTOCd/gVSS∙d for azide-inhibited sludge. First-order kinetics could be used to describe the data with kinetic coefficients of 0.014–0.020 L/gVSS∙d for both the live and azide-inhibited sludge (Table 2). However, using zero-order reaction rates provided equally good fits and the rates of removal shown above were calculated from the linear decrease in TOCd concentrations.

### Fate of small molecules during sorption tests

**Fig 6:**
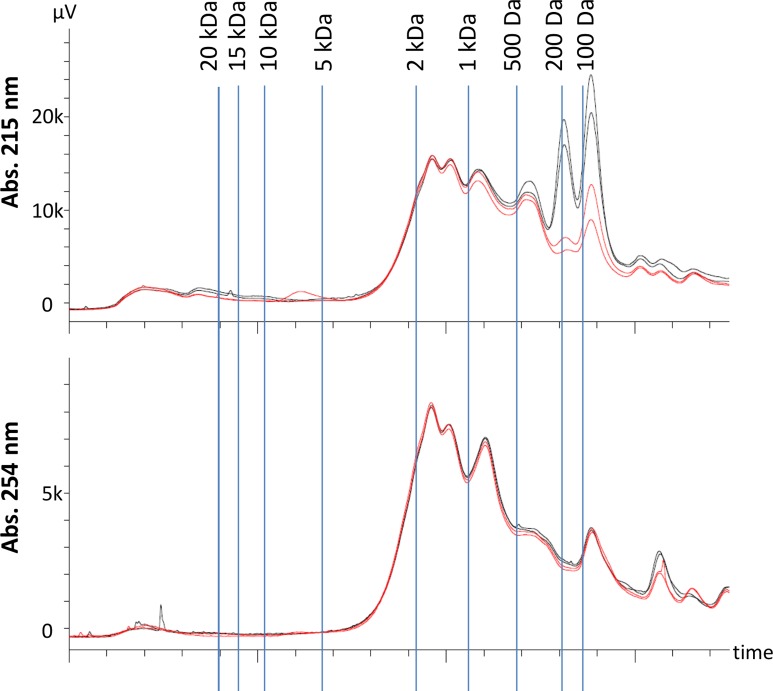
HPSEC of samples from sorption tests with aerobic activated sludge and 1 min mixing. Black lines show controls without addition of sludge. Red lines show samples with addition of 0.97 g/L VSS. The vertical lines show the retention time of polyethylene glycol standards of known molecular weight.

The fate of small molecules (<20 kDa) in the sorption tests was investigated using HPSEC. In this size range, most of the organic molecules were smaller than 2 kDa (Fig. 6). Detection at wavelengths of 215, 230, 254, and 280 nm was carried out. However, the results at 215 and 230 nm were very similar to each other, and so were the results at 254 and 280 nm. Therefore, only the results at 215 and 254 nm are shown in Fig. 6. Organic compounds with functional groups such as carboxyls, carboxylates, aldehydes, ketones, and esters have absorption maxima near 200 nm [25], and these groups are likely detected in the HPSEC spectra at 215 nm. At longer wavelengths, aromatic organic compounds have absorption peaks. Benzene and more complex aromatic molecules such as humic acids have absorption peaks near 254 nm and this wavelength is often used as an indicator of natural organic matter in water [26]. Proteins usually have absorption peaks near 280 nm because of the aromatic amino acids tryptophan and tyrosine [27]. Fig. 6 shows samples after 1 min of mixing, thus the effect of the near-instantaneous sorption event can be observed. At 215 nm, there is a clear difference between sorption tests and controls for molecules of 200 Da and less. This suggests that small molecules such as carboxylic acids may have been rapidly taken up by the sludge upon mixing. At 254 nm, there is no obvious difference between control and sorption test samples for molecules smaller than 20 kDa. As the TOCd values in the kinetic tests were obtained by correlation with ABS254 values, this suggests that the instantaneous sorption of TOCd observed in Fig. 4 is due to dissolved organic matter larger than 20 kDa. The HPSEC spectra obtained after mixing 15 and 120 min are shown in S3 File. For the short wavelengths (215 and 230 nm) the sorption tests samples are still distinctly different from the control around 200 Da at 15 min. However, at 120 min the absorbance at this size range has increased drastically both for the control and test samples, possibly because of fermentation leading to the production of carboxylic acids. At the longer wavelengths (254 and 280 nm), the absorbance of molecules smaller than 200 Da decreases with time in the sorption test samples whereas the control samples remain fairly constant. At 120 min, there is a clear difference between the controls and the test samples. This suggests that prolonged mixing with sludge leads to uptake or conversion of the aromatic fraction of organic molecules smaller than 200 Da.

Analysis with HPLC of low molecular weight organic acids (i.e. formic-, acetic, propionic-, lactic-, butyric-, crotonic-, and valeric acid) showed that neither of these were produced during the sorption tests. Thus, the increase in the UV absorbance (215 and 230 nm) of low molecular weight compounds after 120 min of mixing in the sorption tests was either caused by carboxylic acids larger than C5 or other small organic molecules containing functional groups absorbing at those wavelengths.

## Discussion

Three types of sludges were tested for sorption of organic compounds from raw municipal wastewater. Both primary- and anaerobic sludge had a net release of particles (>0.45 μm). Release of soluble substances (<0.45 μm) was lower; however, increases in the ABS254 values suggested that humic-like substances were released from the sludges. In this study, anaerobic sludge from a completely mixed mesophilic digester was used. It is possible that anaerobic granular sludge could have a net sorption of organics because of different surface characteristics and better settling properties than non-granular anaerobic sludge. Riffat and Dague [28] showed that anaerobic granules could adsorb milk from a synthetic solution. The sorption capacity ranged from less than 10 up to about 45 mgCOD/gTSS. This is similar to the 5-min sorption capacity of aerobic activated sludge observed in our study.

Recirculation of primary sludge to enhance primary sedimentation of wastewater has been suggested as an enhanced primary treatment technology [16]. However, in that study they observed that sorption of organics from the raw wastewater could only be obtained if the sludge was aerated in a separate tank before being contacted with the raw wastewater. Thus, the primary sludge evolved into an aerobic activated sludge. This is consistent with results in our study, which showed that addition of primary sludge did not remove organics from the wastewater, but addition of aerobic activated sludge did.

The 5-min sorption tests with aerobic activated sludge showed average sorption capacities of 6.5±10.8 mgTOCp/gVSS for particulates. Assuming a COD/TOC conversion factor of 2.67 (see S4 File), this is equivalent to 18±29 mgCOD/gVSS. This is similar to the 0–30 mgCOD/gTSS sorption capacity observed by Rensink and Donker [29] for total wastewater COD in 10 min tests and the-40 to 100 mgCOD/gTSS sorption of non-settleable organics observed by Guellil et al. [30] in 60 min tests. The sorption capacity of 5.0±4.7 mgTOCd/gVSS for dissolved organics is also similar to the 0.9–16 mgTOCd/gTSS observed by Jorand et al. [31] in 15-min tests. Variation in sorption capacity between the different tests could depend on variations in both the activated sludge and the wastewater characteristics. The activated sludge appeared to have quite constant characteristics during the experimental period with SVI ranging from 70 to 96 mL/gTSS. The influent wastewater had more variable characteristics, which can be seen by the original values for ABS650, ABS254, TOCp, and TOCd in Figs. 1–3. However, no clear correlations could be found between any single of these parameters and sorption capacity. Instead, it is likely a combination of several different properties of the sludge and the wastewater that determine sorption capacity. Lim et al. [20] showed that operational parameters affecting sludge characteristics such as dissolved oxygen concentration, SRT, and pH can affect sorption capacity.

Sorption kinetics of wastewater organics onto sludge has previously been investigated in very few studies. Jimenez et al. [19] obtained a 1^st^ order rate coefficient (*k*) of about 0.15 L/gTSS·min for non-settlable particulates (>0.45 μm), which can be compared to the 0.05–0.16 L/gVSS·min observed in our study. For organics smaller than 0.45 μm the *k*-values were 0.030–0.049 L/gTSS·min in Jimenez et al. [19] and 0.014–0.065 L/gVSS·min in our study. The observations that both sorption capacity and kinetic coefficients correspond quite well with values obtained from other studies carried out in different countries suggest that the sorption capacity and kinetics of aerobic activated sludge is quite consistent between different treatment plants.

New high-rate activated sludge processes that rely on sorption as a major removal mechanism for organic matter are emerging. For example, Faust et al. [14] operated a high-rate MBR and estimated the degree of mineralization of organics from as low as 1% at 0.2 d to 11% at 1 d SRT. This means that energy can be saved by minimizing the need for aeration and energy can be recovered by maximizing the fraction of the organics that e.g. can be converted to biogas. To be able to model and optimize this and other types of sorption-based process, further knowledge about the mechanisms and rates of sorption is needed. Currently, sorption of organics onto activated sludge is often assumed to be an instantaneous process. For example, this is the assumption made (primarily for particulate and colloidal substrate) in the commonly used activated sludge models developed by the International Water Association [32]. Other studies have pointed out that sorption of organics is not instantaneous but can be modelled using kinetic equations [19], which is especially relevant for high-rate processes operated at short HRTs. In this study, we observed both a near-instantaneous sorption event and a slower sorption process obeying 1^st^ order kinetics. The instantaneous sorption event occurred in less than 1 min of mixing, which was the time for our first sample. Tests with azide-inhibited sludge suggested that neither the instantaneous nor the slower sorption process was coupled to microbial respiration.

Further knowledge is needed about which fractions of the wastewater organics can be sorbed onto activated sludge. In all sorption tests, a significant residual organics concentration was observed in solution. HPSEC results showed that small molecular weight compounds (<200 Da) with strong UV absorbance at 215 and 230 nm were instantaneously taken up by the sludge upon mixing. However, the HPSEC spectra at 254 nm suggested that most of the organic compounds instantaneously sorbed were likely larger than 20 kDa. In a study by Dulekgurgen et al. [33], 43% of the dissolved COD in a municipal wastewater was associated with the size range 10 kDa-0.45 μm. Organics in this size range could include e.g. proteins, polysaccharides and humic acids [34]. After the instantaneous sorption and up to a mixing time of 120 min, the content of aromatic organic compounds smaller than about 200 Da was gradually decreased either by uptake, sorption, or conversion by the activated sludge microorganisms.

## Conclusions

The sorption and release of organic compounds by primary-, anaerobic-, and aerobic activated sludge when mixed with raw municipal wastewater was investigated. Only aerobic activated sludge showed net sorption of organics from the wastewater. Primary- and anaerobic sludge had both net releases of mainly particulate organics.

For aerobic activated sludge, the 5-min sorption capacity was 6.5±10.8 mgTOCp/gVSS for particulate organics and 5.0±4.7 mgTOCd/gVSS for dissolved organics. This is similar to values obtained by other researcher using activated sludge from other treatment plants. Prolonged starvation under aerobic conditions of the activated sludge (>1 d) did not improve sorption capacity, instead it led to deflocculation.

When activated sludge was mixed with wastewater, a near-instantaneous sorption of dissolved organics (<0.45 μm) could be observed followed by a slower sorption obeying 1^st^ order kinetics. For particles, there was an instantaneous release of particles when sludge and wastewater was mixed, followed by sorption according to 1^st^ order kinetics. The kinetic rate coefficient was 0.05–0.16 L/gVSS·min for particles and 0.014–0.065 L/gVSS·min for dissolved organics.

HPSEC results suggested that organic compounds larger than 20 kDa but smaller than 0.45 μm as well as small molecules (<200 Da) with UV absorbance at 215–230 nm were almost instantaneously removed from the wastewater when mixed with activated sludge.

## Supporting Information

S1 File Calculation of velocity gradient in batch tests. (PDF)

S2 File Correlations between absorbance measurements and TSS and TOCd. (PDF)

S3 File High-performance size exclusion chromatography (HPSEC) profiles. (PDF)

S4 File Conversion between COD and TOC. (PDF)
