# Supplementary material for: Sorption and Release of Organics by Primary, Anaerobic, and Aerobic Activated Sludge Mixed with Raw Municipal Wastewater
Source: PLoS One. 2015 Mar 13;10(3):e0119371. doi: 10.1371/journal.pone.0119371 (PMC4359093; doi:10.1371/journal.pone.0119371)
Supplement: S1 File — (PDF) [file pone.0119371.s001.pdf]

Supplementary information file S1

Calculation of velocity gradient in batch tests

The velocity gradient can be calculated using the equation below [1]:

$$G = \left(\frac{\epsilon}{\mu}\right)^{1/2}$$

where *G* is the velocity gradient (s^{-1}), ϵ is the dissipated power per volume ($Nm\ s^{-1}\ m^{-3}$), and μ is the dynamic viscosity of the liquid ($Ns\ m^{-2}$).

The dissipated power per volume can be calculated using the equation below [1]:

$$\epsilon = \frac{P_O \cdot N^3 \cdot D^5}{V}$$

where P_O is the power number, *N* is the impeller speed (rotations per second), *D* is the impeller diameter (m), and *V* is the tank volume (m^3).

For the batch vessels used in this study, the Reynold's number was calculated to 10^4 using the equation below.

$$Re = \frac{\rho \cdot N \cdot D^2}{\mu} = \frac{998.2 \cdot 3.33 \cdot 0.055^2}{0.001} = 10^4$$

where *Re* is the Reynold's number, ρ is the density of water (998.2 kg m^{-3}), *N* is the impeller speed (3.33 rotations per second), *D* is the impeller diameter (0.055 m), and μ is the viscosity of water (0.001 kg s^{-1} m^{-1}).

For cylindrical vessels without baffles, which were used in this study, the power number for paddle impellers at a Reynold's number of 10^4 has been estimated to 0.7 [1]. Using this value for P_O, the dissipated power per volume can be calculated.

$$\epsilon = \frac{P_O \cdot N^3 \cdot D^5}{V} = \frac{0.7 \cdot 3.33^3 \cdot 0.055^5}{0.7 \cdot 10^{-3}} = 1.86 \cdot 10^{-2}\ N\,m\,s^{-1}\,m^{-3}$$

Subsequently, the velocity gradient in the batch vessels used in this study was calculated to 4.3 s^{-1}.

$$G = \left(\frac{\epsilon}{\mu}\right)^{1/2} = \left(\frac{1.86 \cdot 10^{-2}}{0.001}\right)^{1/2} = 4.3\ s^{-1}$$

References

1. Leentvaar J, Ywema TSJ (1980) Some dimensionless parameters of impeller power in coagulation-flocculation processes. Water Research 14: 135-140.
