# Supplementary material for: Sorption and Release of Organics by Primary, Anaerobic, and Aerobic Activated Sludge Mixed with Raw Municipal Wastewater
Source: PLoS One. 2015 Mar 13;10(3):e0119371. doi: 10.1371/journal.pone.0119371 (PMC4359093; doi:10.1371/journal.pone.0119371)
Supplement: S2 File — (PDF) [file pone.0119371.s002.pdf]

Supplementary information file S2

Correlations between absorbance measurements and TSS and TOCd

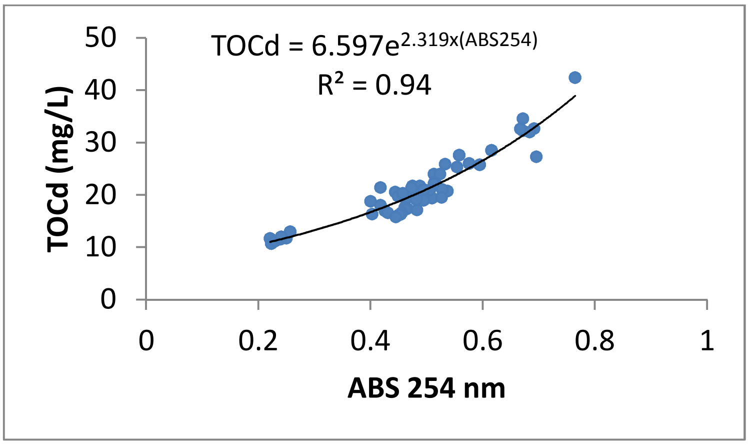

Correlation between dissolved organic carbon (TOCd) and absorbance at 254 nm.

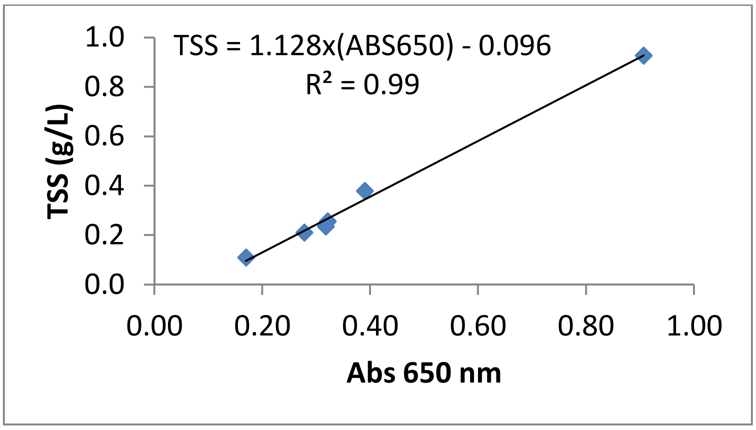

Correlation between total suspended solids (TSS) and absorbance at 650 nm.
