# Supplementary material for: Sorption and Release of Organics by Primary, Anaerobic, and Aerobic Activated Sludge Mixed with Raw Municipal Wastewater
Source: PLoS One. 2015 Mar 13;10(3):e0119371. doi: 10.1371/journal.pone.0119371 (PMC4359093; doi:10.1371/journal.pone.0119371)
Supplement: S3 File — (PDF) [file pone.0119371.s003.pdf]

Supplementary information file S3

High-performance size exclusion chromatography (HPSEC) profiles

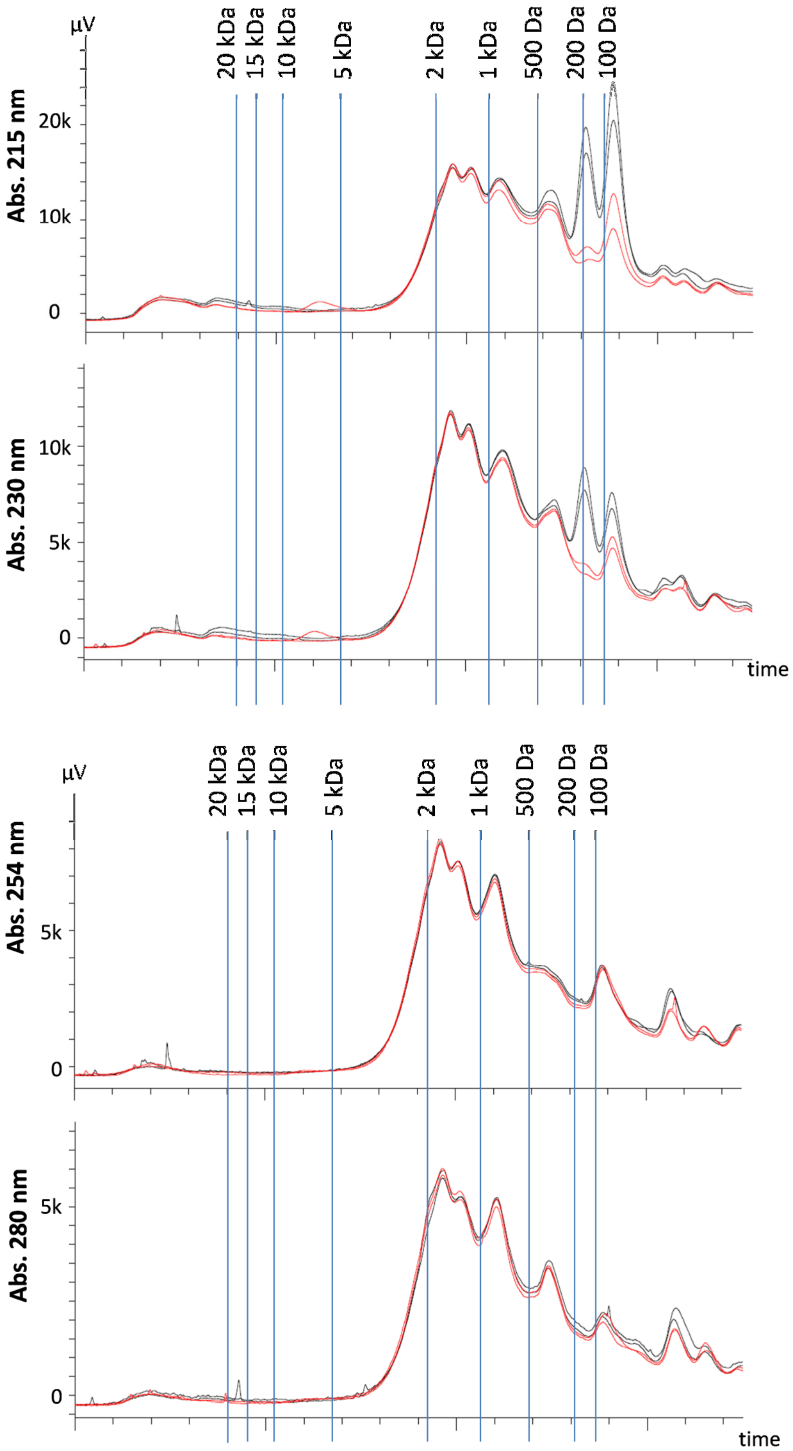

HPSEC profiles after **1 min mixing**. Black lines are controls without added sludge. Red lines are sorption tests with 0.97 g/L VSS added. The vertical lines show the retention time of polyethylene glycol standards of known molecular weight.

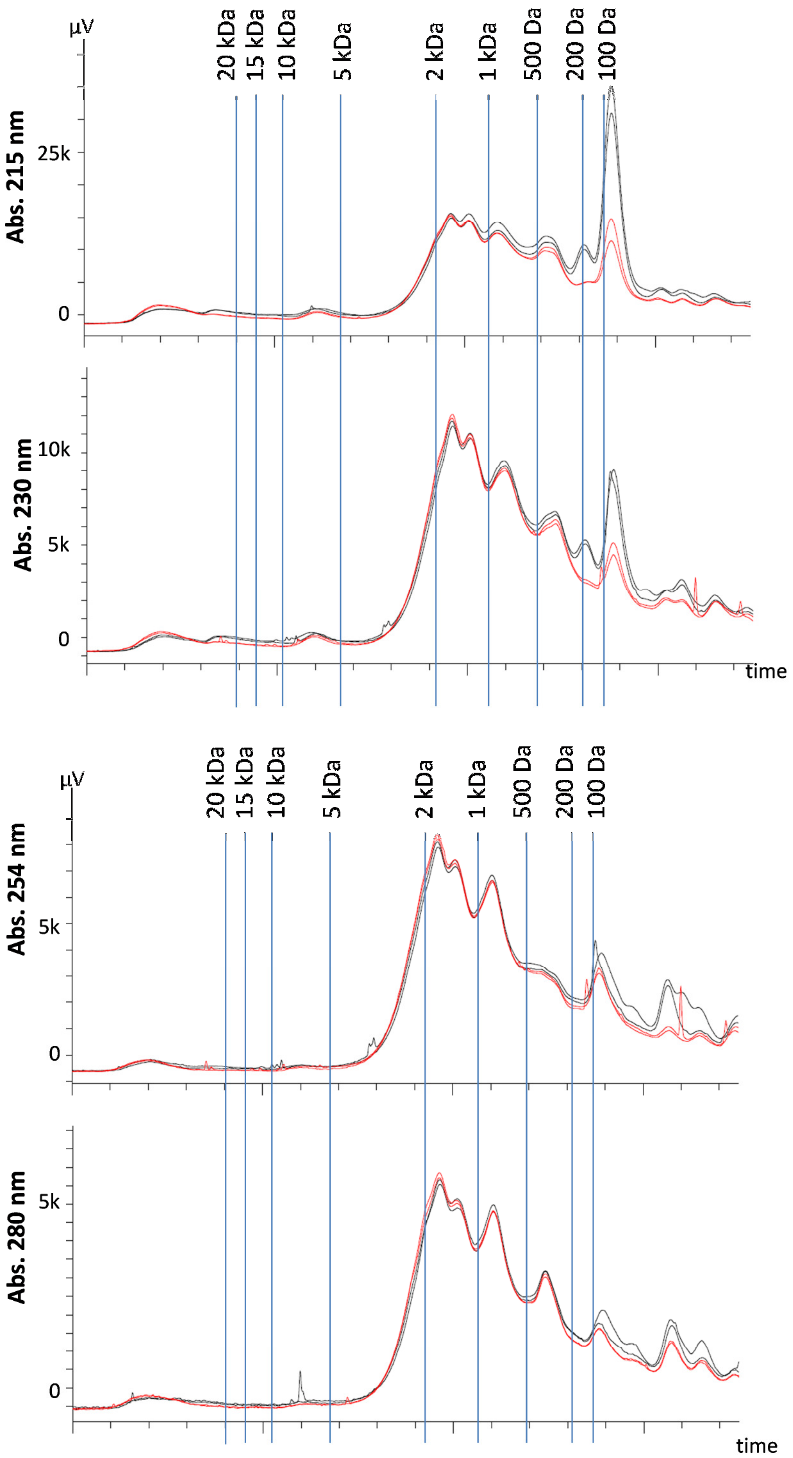

HPSEC profiles after **15 min mixing**. Black lines are controls without added sludge. Red lines are sorption tests with 0.97 g/L VSS added. The vertical lines show the retention time of polyethylene glycol standards of known molecular weight.

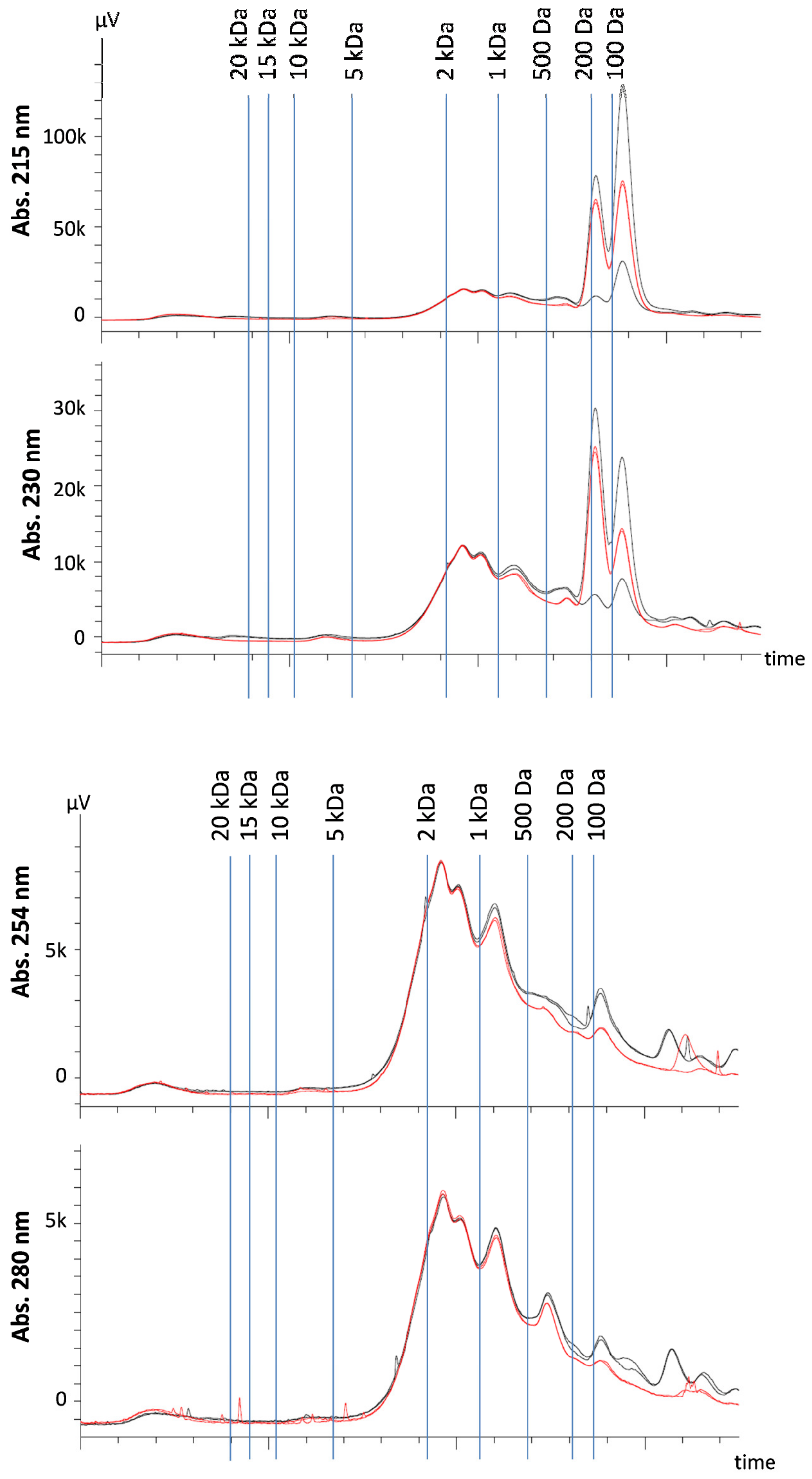

HPSEC profiles after **120 min mixing**. Black lines are controls without added sludge. Red lines are sorption tests with 0.97 g/L VSS added. The vertical lines show the retention time of polyethylene glycol standards of known molecular weight.
