# Supplementary material for: Sorption and Release of Organics by Primary, Anaerobic, and Aerobic Activated Sludge Mixed with Raw Municipal Wastewater
Source: PLoS One. 2015 Mar 13;10(3):e0119371. doi: 10.1371/journal.pone.0119371 (PMC4359093; doi:10.1371/journal.pone.0119371)
Supplement: S4 File — (PDF) [file pone.0119371.s004.pdf]

Supplementary information file S4

Conversion between COD and TOC

The organic matter is assumed to have the elemental composition CH_2O. Then, oxidation takes place according to the equation below.

$CH_2O + O_2 \rightarrow CO_2 + H_2O$

A conversion factor between COD and TOC thus becomes:
32 g O_2 per 12 g C $\rightarrow$ 32/12 = 2.67 gCOD/gTOC
